# Risedronate-loaded aerogel scaffolds for bone regeneration

**DOI:** 10.1080/10717544.2022.2152135

**Published:** 2022-12-06

**Authors:** Nahla El-Wakil, Rabab Kamel, Azza A. Mahmoud, Alain Dufresne, Ragab E. Abouzeid, Mahmoud T. Abo El-Fadl, Amr Maged

**Affiliations:** a Cellulose and Paper Department, National Research Centre, Giza, Egypt; b Pharmaceutical Technology Department, National Research Centre, Giza, Egypt; c Pharmaceutics and Pharmaceutical Technology Department, Faculty of Pharmacy, Future University in Egypt, New Cairo, Egypt; d CNRS, Grenoble INP, LGP2, Université Grenoble Alpes, Grenoble, France; e Biochemistry Department, Biotechnology Research Institute, National Research Centre, Giza, Egypt; f Cancer Biology and Genetics Laboratory, Centre of Excellence for Advanced Sciences, National Research Centre, Giza, Egypt; g Pharmaceutical Factory, Faculty of Pharmacy, Future University in Egypt, New Cairo, Egypt

**Keywords:** Risedronate sodium, cellulose, crosslinking, chitosan, aerogel, implants, cell viability, RUNX2

## Abstract

Sugarcane bagasse-derived nanofibrillated cellulose (NFC), a type of cellulose with a fibrous structure, is potentially used in the pharmaceutical field. Regeneration of this cellulose using a green process offers a more accessible and less ordered cellulose II structure (amorphous cellulose; AmC). Furthermore, the preparation of cross-linked cellulose (NFC/AmC) provides a dual advantage by building a structural block that could exhibit distinct mechanical properties. 3D aerogel scaffolds loaded with risedronate were prepared in our study using NFC or cross-linked cellulose (NFC/AmC), then combined with different concentrations of chitosan. Results proved that the aerogel scaffolds composed of NFC and chitosan had significantly improved the mechanical properties and retarded drug release compared to all other fabricated aerogel scaffolds. The aerogel scaffolds containing the highest concentration of chitosan (SC-T3) attained the highest compressive strength and mean release time values (415 ± 41.80 kPa and 2.61 ± 0.23 h, respectively). Scanning electron microscope images proved the uniform highly porous microstructure of SC-T3 with interconnectedness. All the tested medicated as well as unmedicated aerogel scaffolds had the ability to regenerate bone as assessed using the MG-63 cell line, with the former attaining a higher effect than the latter. However, SC-T3 aerogel scaffolds possessed a lower regenerative effect than those composed of NFC only. This study highlights the promising approach of the use of biopolymers derived from agro-wastes for tissue engineering.

## 1. Introduction

Reactivity and chemical behavior of cellulose are determined by the physical features that originate from the method of its preparation and processing, additionally to its chemical composition. Due to ability to functionalization and compatibility of cellulose, it can be tailored, during processing, to expand its application to match the intended purpose (Nishiyama et al., 2002). Moreover, because of its strong binding intra- and intermolecular hydrogen bonding and partially crystalline structure, cellulosic fibers unable to be melted or dissolved in common solvents and only a few direct solvent solutions exist for cellulose dissolution. The sodium hydroxide/urea system is regarded as the easiest, safest, and fastest of these solvents (Ni et al., 2014; Yang et al., 2017).

Elementary fibrils and less-ordered amorphous areas and crystallites, are the building blocks of cellulosic fibers. Regarding the accessibility and reactivity of the fibers, the amorphous areas and inner surface area of voids are influential. The dissolution of cellulose followed by regeneration is reported to be a successful technique for making amorphous cellulose. Regenerated fibers display a crystalline/amorphous microfibrillar structure, which is independent of the cellulose transformation process into the solution and subsequent fiber production processes (two-phase model) (Deguchi et al., 2008).

The stable hydrogen-bonded networks between the cellulose and the solvent constituents (sodium hydroxide and urea) are formed at low temperature, which is assumed to be influenced by the entropy hydrate formation kinetics and resulted in the breakdown of cellulose. The low temperature requirement for cellulose dissolution prevents the chemical agents from evaporating, making this aqueous system a green and safe solvent in addition to its low cost and availability (Cai et al., 2008; Ni et al., 2014; Li et al., 2015).

In this study we exploit the benefits of two forms of cellulose: amorphous or regenerated cellulose (AmC) and TEMPO-oxidized nanofibrillated cellulose (NFC). AmC resulted from the dissolution of cellulose in sodium hydroxide/urea system followed by regeneration, while NFC are cellulosic fibers that have undergone mechanical disintegration to become a network of cellulose microfibrils. NFC have a nanoscale (less than 100 nm) diameter and a typical length of several micrometers. Due to its strong mechanical reinforcement properties, this bio-based nanomaterial has mostly been used in medical and non-medical applications (Abouzeid et al., 2018, 2019; Kamel et al., 2020).

Chemical or physical crosslinking can be used to generate cellulose-based hydrogels. Physically cross-linked hydrogels are vulnerable that it may be disrupted if the surrounding environment changed (Romano et al., 2007) while chemically cross-linked ones are durable, however, the commonly used crosslinking agents such as epichlorohydrin or glutaraldehyde are toxic (Uliniuc et al., 2013).

According to reports, citric acid is a safe and nontoxic crosslinking agent (Franklin & Guhanathan, 2014; Mali et al., 2018). Citric acid generates a cyclic anhydride and esterifies the hydroxyl groups on the close polymer chains to produce crosslinks at sufficiently high temperatures. Various cellulose derivative systems were prepared using citric acid as a crosslinking agent (Demitri et al., 2008; Hassan et al., 2020) showing interesting and promising outcomes (Coma et al., 2003; Raucci et al., 2015; Bozova & Petrov, 2021). Hence, citric acid was employed as a crosslinker in this study to exploit the advantages of AmC and NFC in a formula intended for bone regeneration.

Cellulose fibers can form aerogel scaffolds of high porosity and low solid content with low weight and density and having a macromolecular open interconnected microstructure (Liebner et al., 2008; Silva et al., 2012). Such biopolymers-based aerogels are extensively utilized in the delivery of various types of therapeutic agents (Kamel et al., 2022a; Khalil et al., 2023).

Extracellular matrix (ECM) made of biodegradable polymers in the form of three-dimensional porous scaffolds have been utilized for in-situ tissue reconstruction (Nair & Laurencin, 2006; Ulery et al., 2011; Elkasabgy & Mahmoud, 2019). The scaffolds must be biocompatible, biodegradable, with large specific surface area; in addition to high porosity with uniform interconnected pores to allow sufficient transport of nutrients. Also, good mechanical properties are essential to match with a degradation or resorption rate convenient for remodeling and tissue replacement (Morrison, 2009; Vinatier et al., 2009). A uni-polymeric scaffold cannot impart all the needed characteristics, using a polymeric blend can allow to tailor a scaffold to attain the desired characteristics (Gautam et al., 2013; Jafari et al., 2017; Elkasabgy et al., 2019), therefore, polymer blended scaffolds are necessary for tissue reconstruction (Joshi et al., 2013). The combination between chitosan and cellulosic derivatives has proved a great success for such an application (Maged et al., 2019).

Chitosan (CS) is a high molecular weight cationic biocompatible polysaccharide derived from chitin which is obtained from the shellfish exoskeleton. Chitosan is widely applied in tissue engineering as well as other biomedical applications (Crini & Badot, 2008; Cheng et al., 2015; Lupascu et al., 2015; Reys et al., 2017; Abdel-Salam et al., 2020). It is a weak base soluble in acidic solution due to protonation of the free amino group (pH < 6.5) (He et al., 2011). Chitosan can stimulate the activity of growth factors (Ueno et al., 2001) and can aid the maintenance of the chondrogenic phenotype (Sechriest et al., 2000), hence it was successfully used as a tissue culture scaffold and healing dressing (Noel et al., 2008; Croisier & Jérôme, 2013; Elsabee & Abdou, 2013; Castro & Paulín, 2015; Nitta et al., 2015; Ma et al., 2017).

Risedronate sodium is a vital drug belonging to the biophosphonate family and used mainly in bone regeneration. This drug is a white to off-white crystalline powder with a molecular weight of 305.09 Da (O’Neil, 2001). The high water solubility (16.5 mg/mL) and low oral bioavailability (0.63%) (Mitchell et al., 2001) of risedronate classify it in BCS as class III drug. The incorporation of risedronate into biomaterials-based drug delivery systems revealed synergetic results in the field of bone regeneration (Elkasabgy et al., 2018; Khajuria et al., 2018; Mostafa et al., 2021).

In this study, the fabricated 3D aerogel scaffolds were used as a carrier for risedronate. Bisphosphonates are therapeutic agents used for the treatment of bone disorders. The regular turnover of bone is maintained by a balance between the actions of osteoblasts (cells that build bone) and osteoclasts (bone destroying cells). Bisphosphonates decrease bone loss by inhibiting the action of osteoclasts (Weinstein et al., 2009). Loading the scaffolds with a drug inhibiting bone resorption like bisphosphonates can optimize the treatment of bone defects. Therefore, cellulose-based 3D aerogel scaffolds, loaded with risedronate, were prepared by chemical cross-linking (with amorphous cellulose) or physical cross-linking (with chitosan), and they were evaluated for their physicochemical and cell regenerative properties.

## 2. Materials and methods

### 2.1. Materials

Bleached bagasse Kraft Pulp was kindly delivered by Qena Paper Industry Company, Egypt. The chemical composition of bagasse pulp was 71% α-cellulose, 30% pentosans, 0.8% ash, and a degree of polymerization (DP) of 1200. Risedronate sodium was kindly supplied by Hikma Pharmaceutical and Chemical Industries Company, Egypt. Chitosan low molecular weight, NaOH, urea, NaBr, NaClO, 2,2,6,6-tetramethylpiperidine-1-oxyl radical (TEMPO) were purchased from Sigma-Aldrich, Germany. Citric acid monohydrate was purchased from Riedel-deHaen, Germany. Fetal bovine serum (FBS), L-glutamine, penicillin G sodium, streptomycin sulfate, and amphotericin B were obtained from Lonza, Basel, Switzerland. MTT (3-[4, 5-dimethylthiazole-2-yl]-2,5-diphenyltetrazolium bromide), acridine orange and ethidium bromide were obtained from Merck, Darmstadt, Germany.

### 2.2. Methods

#### 2.2.1. Preparation of amorphous (regenerated) cellulose nanofibers (AmC)

An aqueous solution mixture containing NaOH, urea, and distilled water (7:12:81 by weight) was prepared in a 250 mL beaker. To produce a clear cellulose solution with a concentration of 3%, the aqueous solution was precooled to -12 °C, and cellulose pulp was added to it. The resulting cellulose solution was stored in a refrigerator at 4 °C for 48 hours. Cellulose fibers were regenerated from the cellulose solution through coagulation. The coagulant was formed by adding water to the aqueous solution and then filtration (El-Wakil & Hassan, 2008). The regenerated fibers were washed with distilled water. The regenerated cellulose was disintegrated using a Masuko grinder (Masuko Sangyo Co., Ltd., Japan) at 1500 rpm while varying the gap between the rotating disks and running the fibers through it for 120 minutes (El-Wakil et al., 2022).

#### 2.2.2. Tempo-oxidized cellulose nanofibers (nanofibrillated cellulose; NFC)

TEMPO-assisted oxidation of cellulose fibers was used to prepare surface-functionalized nanofibrillated cellulose (NFC) (Patiño-Masó et al., 2019). Cellulose fibers (10 g) were suspended in 750 mL of water containing 0.025 g of TEMPO and 0.25 g of NaBr. The slurry was then enhanced with NaClO solution (3.84 mmol/g of cellulose) while being continuously stirred. NaOH (0.5 N) was used to maintain the suspension’s pH at 10.5 at room temperature. Finally, the reaction was stopped, and HCl was used to adjust pH to neutrality. The never-dried oxidized cellulose was defibrillated using a Masuko grinder.

#### 2.2.3. Preparation of cross-linked cellulose (NFC/AmC)

The prepared regenerated (AmC) and TEMPO-oxidized cellulose nanofibers (NFC) (1:1 w/w) were subsequently soaked in 20 w % citric acid solution in the presence of 1 w % sodium hypophosphate and autoclaved at 120 °C for one hour. The cured sample was dialyzed in water to eliminate unreacted compounds before being freeze dried (Mali et al., 2018).

#### 2.2.4. Characterization of the prepared cross-linked cellulose

##### 2.2.4.1. Fourier transform infrared spectroscopy (FTIR)

FTIR spectrophotometer (Model 22, Bruker, Alpha II, UK) with a spectrum range between 400 cm^- 1^ and 4000 cm^-1^ was used to detect the spectra of the samples.

##### 2.2.4.2. Differential scanning calorimetry (DSC)

The samples were subjected to a DSC analysis using a DSC131 Evo, manufactured by SETARAM Inc. in France. The purging gas utilized was nitrogen. The temperature range used for the test was -30 to 450 °C, and the samples were heated at a rate of 10 °C per minute. The sample was added to the DSC after being put in a 120 µL aluminum crucible. Results of the thermogram were obtained by employing CALISTO Data processing software v.149.

##### 2.2.4.3. X-ray diffraction (XRD)

X-ray diffraction patterns were recorded using an PANalytical X-ray diffractometer (Netherlands) with a monochromatic Cu K radiation source (0.5418 A) in angle from 4° to 60° at room temperature. Using the Segal method, the crystallinity index was calculated (Segal et al., 1959).

(1) Crystallinity index=(1−(Iam)/(I200))

In this equation, I200 represents the maximum intensity of the crystalline peak located at 2θ = 22-23°, while Iam represents the intensity of the valley associated with the amorphous material located at 2θ = 18-19°.

##### 2.2.4.4. Scanning electron microscope (SEM)

The micro-morphological structure of the samples was examined using scanning electron microscopy (SEM; JSM-6400; JEOL, Tokyo, Japan) at 5–10 kV. Coating with gold was done by sputter coater system (Edwards Sputter Coater, UK).

#### 2.2.5. Preparation of risedronate loaded- aerogel scaffolds

The fabricated aerogel scaffolds were prepared from NFC and NFC/AmC as mentioned in Table 1. The drug (10 mg/aerogel scaffold) was stirred with cellulose matrix using a VELP magnetic stirrer (AREC.T, Italy) at room temperature for 30 minutes to complete the distribution of the drug into the cellulose matrix.

**Table 1.** Risedronate-loaded aerogel scaffolds’ composition.

Formulation code	Composition			Compressive strength (kPa)	Mean release time (h)	Release efficiency (%)
	Cellulosic matrix	Chitosan (%w/v)	Citric acid (%w/v)			
SC-T	NFC	0.000	0.000	20 ± 2.02	0.49 ± 0.04	95.45 ± 2.67
SC-C	NFC/AmC	0.000	0.000	180 ± 18.80	0.50 ± 0.04	90.15 ± 2.43
SC-T1	NFC	0.900	0.450	240 ± 22.30	0.46 ± 0.04	94.80 ± 1.70
SC-T2		1.250	0.625	300 ± 30.60	1.69 ± 0.00	84.69 ± 2.10
SC-T3		2.500	1.250	415 ± 41.80	2.61 ± 0.23	82.17 ± 2.04
SC-C1	NFC/AmC	0.900	0.450	200 ± 13.80	0.61 ± 0.01	94.36 ± 3.08
SC-C2		1.250	0.625	210 ± 18.90	0.64 ± 0.03	93.08 ± 2.12
SC-C3		2.500	1.250	215 ± 15.80	0.58 ± 0.03	94.29 ± 1.56

Each value represents the mean ± SD (*n* = 4).

NFC: Nanofibrillated cellulose (solid content 1.8% w/v).

NFC/AmC: Nanofibrillated cellulose chemically cross-linked (solid content 1.8% w/v).

For aerogel scaffolds containing chitosan, citric acid was dissolved in distilled water using magnetic stirrer for 10 minutes and then, the chitosan was added and stirred for 30 minutes at 40 °C. Our preliminary study showed that to dissolve chitosan in the aqueous solution it needs citric acid in 2:1 weight ratio of chitosan to citric acid. After getting a clear chitosan solution, it was added stepwise into the cellulose matrix under stirring.

After reaching a homogenous solution, it was poured into a specific rounded blister mold (15 × 5 mm for diameter x height) and frozen at - 80 °C for two days. After that, samples that had been frozen were lyophilized using Christ freeze dryer (Alpha 2-4 LD plus, Germany).

#### 2.2.6. Characterization of risedronate loaded- aerogel scaffolds

##### 2.2.6.1. Friability test

Regarding the United States Pharmacopeia (USP), the physical stability of the fabricated aerogel scaffolds was tested using Pharma Friability Tester (PTF ERA, Germany). The aerogel scaffolds were installed in the friabilator’s drum and adjusted to rotate for 4 minutes using 25 rpm as a rotation speed (The United States Pharmacopoeia, 2016). The following equation is used to calculate the % weight loss of scaffolds:

$$\%\;\mathrm{weight}\;\mathrm{loss}=\;\frac{\mathrm{Initial}\;\mathrm{weight}-\mathrm{Final}\;\mathrm{weight}\;}{\mathrm{Final}\;\mathrm{weight}}\times 100$$

where, a value of less than 1% for % weight loss is necessary to pass the test.

##### 2.2.6.2. Compressibility testing

The TAPPI T494-06 standard method was used to evaluate the compressive strength of the aerogel scaffolds utilizing a universal testing machine (LR10K; Lloyd Instruments, Fareham, UK) equipped with a 20-N load cell and a constant crosshead speed of 2.5 cm/min.

##### 2.2.6.3. Porosity

Using a digital balance, the dry weight (Wd) of the freeze-dried aerogel scaffolds was determined (Sartorius AG, Germany). Additionally, a helium pycnometer was used to quantify the bulk volume (Vb) (UltraPyc 1200e, model 2014, Quantachrome, USA). The following equation was used to compute the bulk density (ρb):

$$\rho b\left(g/{\text{cm}}^{3}\right)=\text{Wd}/\text{Vb}$$

The true density (ρg, g/cm^3^) was estimated at 25 °C and 19 psi pressure by the helium pycnometer (UltraPyc 1200e, model 2014, Quantachrome) and then the porosity was calculated based on the following equation (Danielson & Porosity, 1986):

∅%=100*(ρg−ρb)/ρg

##### 2.2.6.4. In-vitro drug release

The *in-vitro* drug release of risedronate from the fabricated aerogel scaffolds was evaluated by immersing the aerogel scaffolds in 5 mL solution of phosphate buffer saline (pH 7.4) in a well-closed glass jar. The glass jar was shaken at a rate of 150 rpm using an IKA incubator shaker (KS 4000, Germany) with an adjusting temperature at 37 °C. At different time intervals, samples (1 mL) were taken and replaced with the same volume of phosphate buffer to keep the release medium volume constant. At a wavelength of 262 nm, the concentration of risedronate in each sample was measured using Shimadzu UV spectrophotometer (UV1800, Japan). Release profiles were plotted and model independent release parameters, the mean release time (MRT) (Podczeck, 1993) and the release efficiency (RE) (Khan & CT, 1972), were calculated.

##### 2.2.6.5. Differential scanning calorimetry (DSC)

Differential scanning calorimetry study was performed on selected the aerogel scaffold as well as its individual components, as mentioned previously, except that the test was performed at the heating range from 30 °C to 250 °C with a heating rate of 10 °C/min for samples.

#### 2.2.7. Cell biology study

##### 2.2.7.1. Cells

Human bone osteosarcoma cell line (MG-63) was purchased from ATCC, USA. The Dulbecco’s Modified Eagle’s Medium (DMEM) was used to routinely cultivate the cells. Exactly, 10% fetal bovine serum (FBS), 2 mM L-glutamine, 100 units/mL penicillin G sodium, 100 units/mL streptomycin sulfate, and 250 ng/mL amphotericin B were added to the medium as supplements. The previous items were all purchased from Lonza (Basel, Switzerland). Cells were kept at sub-confluency in humidified air with 5% CO_2_ at 37 °C. After being treated with trypsin/EDTA at 37 °C, monolayer cells were extracted for sub-culturing. Cells with a confluency of 75% were used. Except when otherwise noted, every experiment was performed three times.

##### 2.2.7.2. MTT Cytotoxicity

The MG-63 cell line was used to examine the cytotoxicity of the tested materials using the MTT (3-[4,5-dimethylthiazole-2-yl]-2,5-diphenyltetrazolium bromide). This test is based on the fact that living cells’ active mitochondrial dehydrogenase enzyme can cleave the yellow MTT’s tetrazolium rings into dark blue, insoluble formazan crystals. The crystals are then dissolved, releasing a dark blue color that is inversely proportionate to the quantity of living cells. Briefly, in a 48-well microplate with a flat bottom, cells (1x10^5^ cells/400 L/well) were seeded in serum-free medium and exposed to equal amounts of the test samples weighing around 0.5-0.75 mg for 72 hours at 37 °C in a humidified 5% CO_2_ atmosphere. The MTT solution was added to the medium at a final concentration of 0.5 mg/mL and incubated for an additional 4 hours. Exactly, 250 µL of acidified isopropanol was used to dissolve the formazan crystals. Then photometric determination of the absorbance at 570 nm was applied using microplate ELISA reader (Hansen et al., 1989) (FLUOstar OPTIMA, BMG LABTECH GmbH, Ortenberg, Germany). For each concentration, three times of repeats were carried out, and the average was computed.

##### 2.2.7.3. Mode of cell death

Equal amounts of the samples were inserted in MG-63 cells cultivated on 8 well cell culture slides (SPL, Seoul, South Korea) at a density of 10^4^ cells/well. After 72 hours of incubation, the cells were stained with dual stains of acridine orange (100 μg/mL) and ethidium bromide (100 μg/mL) that were dissolved in phosphate buffer saline (PBS) at equal volumes to examine the mode of cell death. The stained cells were examined using fluorescence microscope (AxioImager Z2, Zeiss, Jena, Germany). Green-colored cells were classified as ‘living’, whereas yellow, orange, or red cells were classified as ‘early apoptotic’, ‘late apoptotic’, or ‘necrotic’, respectively (Leite et al., 1999). Three separate experiments were conducted (*n* = 3).

##### 2.2.7.4. The RUNX2 protein expression

The samples were added in equal amounts to MG-63 cells cultivated on 8-well cell culture slides (SPL, Seoul, South Korea) at a density of 10^4^ cells per well. Then, the cells were fixed with 4% paraformaldehyde and permeabilized with 0.1% tritonX-100 after 72 hours of incubation. The cells were then blocked with 1% bovine serum albumin and incubated overnight at 4 °C with the anti- Runt-related transcription factor 2 (RUNX2) antibody ab192256 (Abcam, Cambridge, United Kingdom) at dilution 1/500. Then, the cells were well washed before being treated with 1/1000 dilution of the secondary antibody goat against rabbit IgG (Alexa Fluor^®^488) ab150077 (Abcam, Cambridge, United Kingdom). After washing and air drying, the glycerol mounting medium with 4′,6-diamidino-2-phenylindole (DAPI) (ab188804, Abcam, Cambridge, United Kingdom) was used as an anti-fade and as a counterstain for the nucleus. The fluorescence intensity of the cells was then evaluated using fluorescent microscope AxioImager Z2 (Zeiss, Jena, Germany) operated using Zen11 Blue edition software.

## 3. Results and discussion

### 3.1. Characterization of the prepared cross-linked nanocellulose

**Figure 1.**
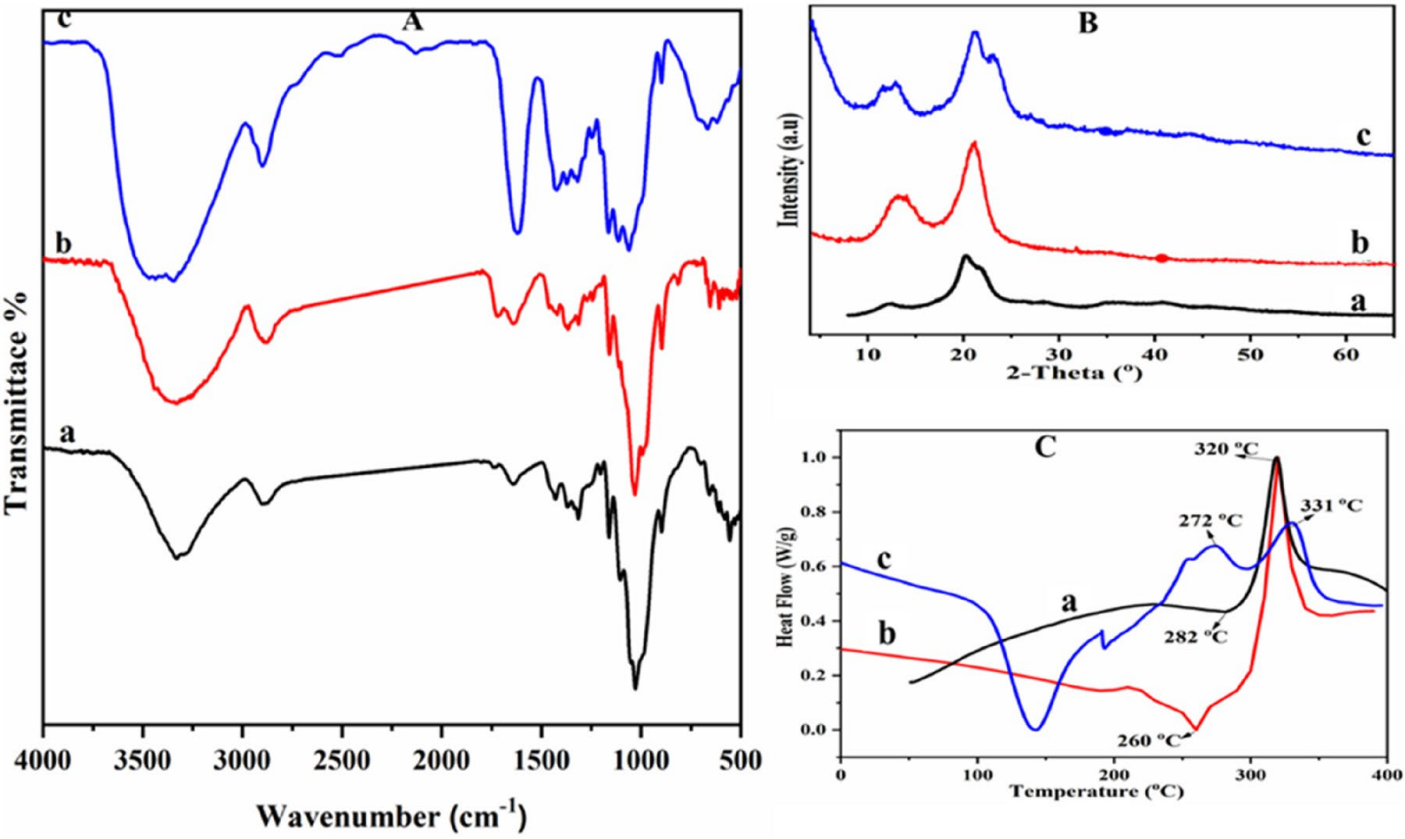
FTIR (A), XRD (B) and DSC (C) of AmC (a), NFC (b) and cross-linked celluloses (NFC/AmC) (c).

FTIR spectra (A), XRD diffraction patterns (B) and DSC thermograms (C) of AmC (a), NFC (b) and cross-linked cellulose (NFC/AmC) (c) are collected in Figure 1 . All FTIR spectra approve the characteristic peaks of cellulose, where the hydroxyl group’s stretching vibration appears at 3330 cm^−1^ which includes inter- and intra-molecular hydrogen bond, while that at 2900 cm^−1^ is assigned to C-H stretching and the peak at 1400 cm^−1^ is assigned to O-H and C-H bending. Also, the peak at 1108 cm^−1^ is due to C-O-C and C-O-H asymmetric stretching. The new peak appearing at 1730 cm^−1^ in spectrum C ascribed to carbonyl C = O stretching reveals the esterification of cellulose due to crosslinking with citric acid.

Figure 1B displays the XRD patterns of the three cellulose (a) AmC, (b) NFC and (c) cross-linked cellulose (NFC/AmC). A cellulose I pattern can be observed for NFC showing the diffraction peaks around 2θ 15.9° and 22.3° assigned to diffused (110), (11̅0) and (200) planes, respectively (Ioelovich, 2013) with a crystallinity of 75%.

In contrast to NFC, AmC’s diffractogram shows cellulose II crystalline structure with diffraction peaks of 12.3°, 19.9°, 21.8° corresponding to (11̅0), (020), and (200) and a crystallinity of 37% which is clarified by the clear reduction in the amorphous peak due to the increased accessibility of the amorphous domains of cellulose after dissolution with sodium hydroxide and urea followed by regeneration (Samayam et al., 2011; Udoetok et al., 2018a). On the other hand, cross-linked cellulose (NFC/AmC) shows diffraction peaks at 10.9°, 12.2°, 19.7°, and 21.4° and a crystallinity of 59%. The XRD patterns display combined features of both NFC and AmC where the diffraction patterns of both are noted demonstrating that the fundamental cellulose structural elements and crystalline domains have been effectively conserved (Udoetok et al., 2018a).

Figure 1C shows the DSC thermogram of NFC, AmC, and cross-linked cellulose (NFC/AmC) recorded from −30 to 450 °C. One endothermic peak due to melting is observed at 260 and 282 °C for NFC and AmC, respectively. During regeneration, it was expected that the AmC would show a lower endothermic transition temperature than other celluloses (Liu et al., 2011; Yeng et al., 2015). However, here AmC did not show a melting peak; this may be ascribed to the further defibrillation step after regeneration which enhanced the entanglement between the fibers and consequently increased the stability. Moreover, a broad endothermic peak appeared at 140 °C for cross-linked cellulose may be due to the evaporation of bound water (Udoetok et al., 2018b) accompanied by the disappearance of melting endothermic peak after the formation of new bonds as a result of crosslinking. This DSC profile is in agreement with that reported by Udoetok et al. (Udoetok et al., 2018b). NFC and AmC showed one exothermic peak at 320 °C due to crystallization, while cross-linked cellulose showed two exothermic peaks at 272 and 331 °C due to the difference in the mode of crystallization of both cross-linked celluloses (AmC and NFC).

**Figure 2.**
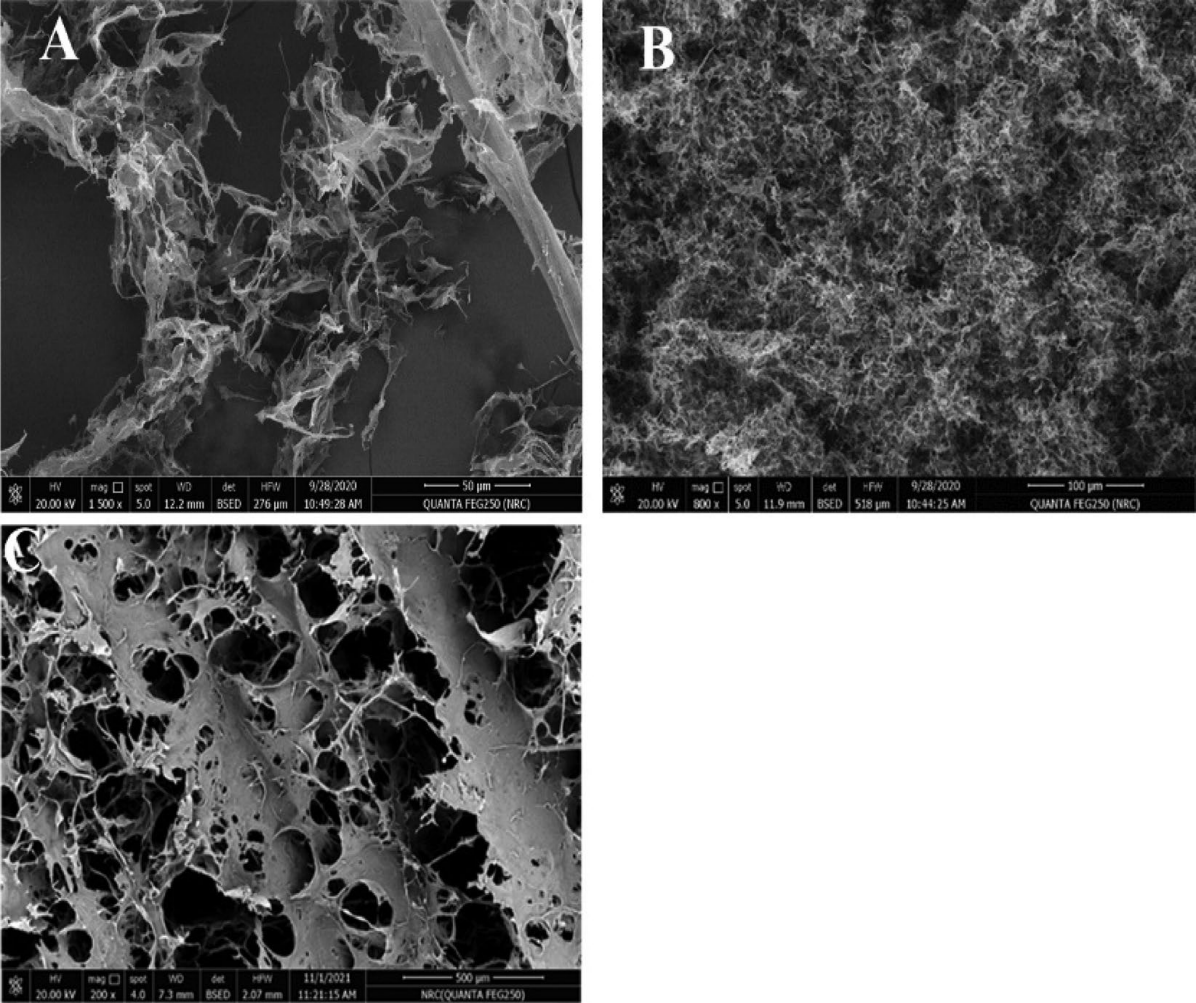
SEM images of AmC (A), NFC (B) and cross-linked cellulose NFC/AmC (C).

Figure 2 illustrates the SEM images of AmC, NFC and cross-linked cellulose (NFC/AmC) denoted as A, B and C, respectively. The SEM picture of AmC reveals some agglomerated structure brought on by hydrophobic interactions as a result of cellulose’s regeneration and subsequent conversion to cellulose II. This reorganization of the intra- and intercellular structure is the outcome of cellulose’s dissolution and regeneration (El-Wakil et al., 2022). The SEM image of NFC demonstrates the dense network structure of the nanofibers with good fibrillation and homogeneity with well-connected pores indicating the readily available structure for loading and modification. Figure C displays the consistent, open and macroporous structure of the cross-linked cellulose (NFC/AmC) allowing for solutes flow and transport of nutrients.

NFC and cross-linked cellulose (NFC/AmC) were selected to formulate drug-loaded aerogel scaffolds due to their dense and porous structure indicating probable good mechanical properties.

### 3.2. Characterization of risedronate loaded- aerogel scaffolds

#### 3.2.1. Friability test

Comparing the NFC (SC-T) and chemically cross-linked NFC/AmC (SC-C) aerogel scaffolds, the former aerogel scaffold composed of TEMPO-oxidized nanofibrillated cellulose succeeded to pass the test without any broken fragments or scaffold cracking, where the % weight loss equals 0%, while the latter failed to pass the test (the % weight loss equals 2.29%), this may be due to the formation of a more rigid structure with low flexibility causing its high brittleness. Furthermore, friability for the aerogel scaffolds depended on the microstructure of the used nanofibers as detected in the above SEM photographs. NFC scaffolds showed excellent mechanical properties where the fibers are arranged tight together in a dense cohesive structure to from stable aerogel scaffolds with low friability compared to those prepared using NFC/AmC.

For the NFC aerogel scaffolds (SC-T), the addition of chitosan in concentrations of 0.9% (SC-T1), 1.25% (SC-T2), and 2.5% (SC-T3) did not affect the friability where they passed the test (% weight loss values equal 0%). Although the addition of chitosan increased the weight of the prepared aerogel scaffold, the integrity was not impaired due to the strong ionic interaction between carboxyl groups of cellulose and amino group of chitosan, resulting in tight and flexible 3D aerogel scaffolds.

The addition of the same concentrations of chitosan to the chemically cross-linked NFC/AmC (SC-C) aerogel scaffolds showed percentage weight loss values of more than 1% (the % weight loss values ranging from 1.58% to 3.17%) for SC-C1, SC-C2, and SC-C3 aerogel scaffolds. These results may be attributed to the lower ionic interaction with chitosan due to the occupation of the active site of the cellulosic matrix after carboxylation by the chemical cross-linking.

#### 3.2.2. Compressibility study

Opposition of a certain material to the direct pressure resulting from compression is known as the compressive strength (Marković et al., 2016). Brittle and tough materials are not ideal candidates for the preparation of implantable scaffolds because of their poor mechanical properties (Kamel et al., 2020). The advantages of the use of cellulose nanofibers, in combination with other biopolymers, for implantation and tissue regeneration were discussed in details in our previous research paper (Kamel et al., 2022a).

SC-T aerogel scaffolds composed of NFC alone were light fluffy aerogel scaffold while those prepared using the chemically cross-linked NFC/AmC (SC-C) had a tighter and more rigid 3D structure sufficient to withstand physical collapse upon compression and handling, hence the compressive strength of the latter increased significantly as listed in Table 1.

Also, addition of chitosan to NFC resulted in the formation of a more cohesive structure causing the amelioration of the mechanical properties due to the chitosan/NFC strong oppositely charged inter-macromolecular interaction. Also, the hydrogen bonding between chitosan and NFC was confirmed in a previous study based on FTIR (Rizal et al., 2021). The compressibility significantly increased by increasing chitosan concentration, SC-T3 aerogel scaffolds attained the significantly highest compressive strength (415 ± 41.80 kPa); this may be due to the greater inter-biopolymeric interaction in addition to the higher total solid content.

On the other side, addition of chitosan to the cross-linked NFC/AmC aerogel scaffolds didn’t cause a significant change in the formed aerogel scaffolds compressibility, this can be expected due to the occupation of the cellulosic active sites by cross-linking, hence the formation of new physical interaction with chitosan was minimal.

#### 3.2.3. Porosity measurement

The tested aerogel scaffolds showed high porosity values (above 90.07 ± 5.82%), which can be expected because of using the freeze drying preparation technique (Savjani et al., 2012). This high degree of porosity can allow for the scaffolds to absorb body interstitial fluids in case of tissue injury and also facilitate the exchange of nutrients and elimination of wastes (Shamma et al., 2017). A previous study claimed that the ability to respond to, and to withstand to applied compression forces was improved by increasing porosity (Xie et al., 2006), hence, the highly porous structure is a an additional advantage for implantable preparations.

#### 3.2.4. In-vitro drug release

**Figure 3.**
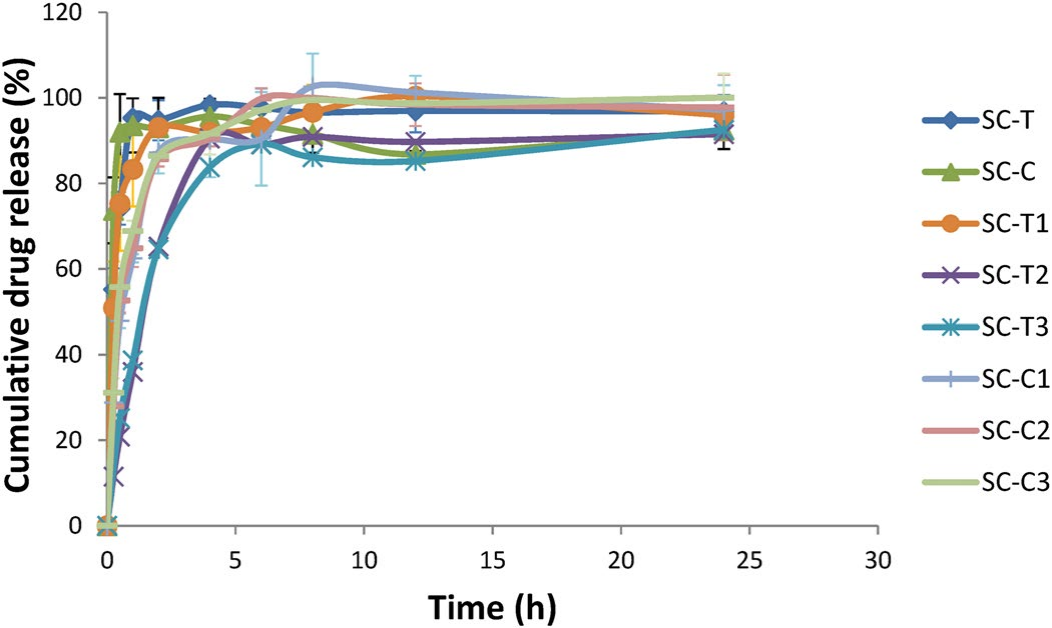
Cumulative drug release profiles for risedronate loaded- aerogel scaffolds.

Figure 3 and Table 1 represent the release patterns and parameters for risedronate from the tested aerogel scaffolds.

The release profiles of the aerogel scaffolds composed of cellulosic matrix without the addition of chitosan (SC-T and SC-C) did not show any significant difference in MRT values and RE values (*p* > 0.05). An almost complete drug release was attained within 1 h, this abrupt drug release is attributed to the high water solubility of risedronate (Elnaggar et al., 2019), resulting in rapid drug release from the aerogel scaffolds upon contact with the release medium. On the other hand, the high porosity of the aerogel scaffolds and leaching of drug molecules from the outer layers can cause the rapid initial drug release (burst effect) (Kamel, 2013).

Aerogel scaffolds formed by the addition of chitosan to NFC in the concentration of 1.25% (SC-T2) and 2.5% (SC-T3) revealed a significant difference (*p* < 0.05) in MRT and RE compared to SC-T aerogel scaffold as well as SC-T1 aerogel scaffold prepared with 0.9% chitosan, where SC-T2 and SC-T3 aerogel scaffolds succeeded to slow down drug release up to 24 hrs., this can be expected due to the strong ionic interaction occurring between chitosan and NFC forming a cohesive matrix and causing the entanglement of the fibrous network, which in turn allowed for the capture of drug molecules within the matrix. Also, one of the privileges of having chitosan incorporated in the scaffold is the formation of a jellified viscous structure upon contact with the release medium (Maged et al., 2019), the scaffold matrix starts to absorb water and swell; increasing the chitosan concentration can increase the swelling degree and the time for the drug to escape out of the tortuous channels created within the matrix resulting in the extension of drug release (Ammar et al., 2009; Kamel & Abbas, 2013).

On the other hand, the addition of the same concentrations of chitosan to the cross-linked NFC/AmC aerogel scaffolds (SC-C1, SC-C2, and SC-C3) did not reveal any significant difference in drug release profiles and parameters compared to that of SC-C aerogel scaffold (*p* > 0.05). This may be attibuted to the low chitosan/cellulose interaction due to the full occupation of the active sites of the cross-linked mixed cellulosic matrix; consequently, the drug was rapidly released.

In summary, the SC-T3 aerogel scaffold with the significantly best mechanical properties (0% friability and highest compressive strength) and most extended drug release (highest MDT value) compared to all other prepared aerogel scaffolds (*p* < 0.05) was chosen for further investigations.

#### 3.2.5. Differential scanning calorimetry (DSC)

DSC is a vital thermal analysis test that study the changes occurring in the thermograms of individual components compared to that of the fabricated scaffold, these changes indicate the formation of a new structure. Rizal and his team prepared aerogel implants containing chitosan and NFC, the DSC analysis of individual components revealed their thermal decomposition at lower temperatures compared to that recorded for the implants due to strong interaction between the components (Rizal et al., 2021).

**Figure 4.**
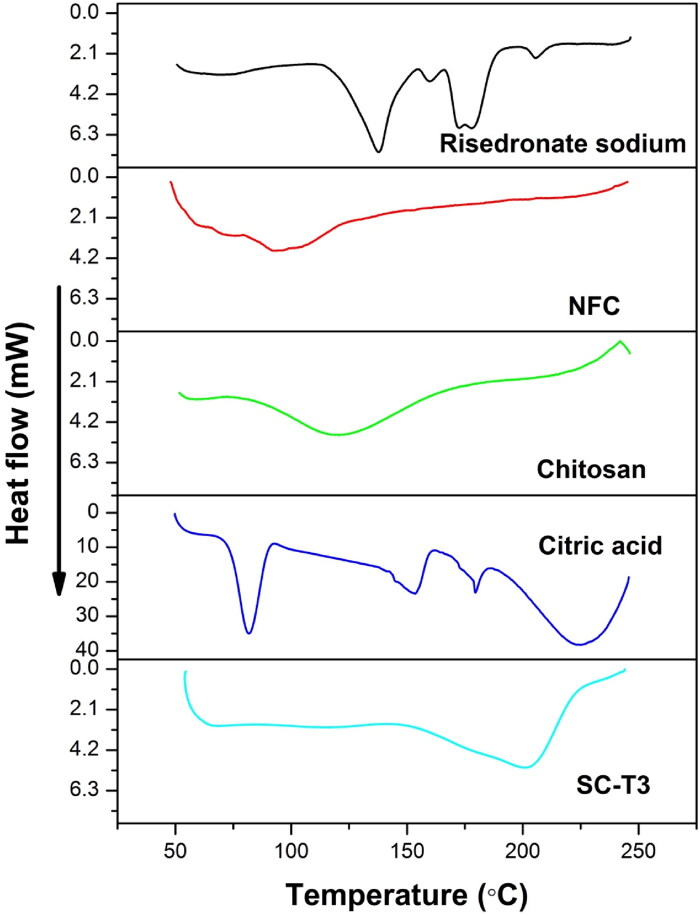
DSC thermogram of risedronate sodium, NFC, chitosan, citric acid and the selected aerogel scaffold SC-T3.

Figure 4 shows the thermal analysis of risedronate, NFC, chitosan, citric acid, and SC-T3 aerogel scaffold. The DSC thermogram of risedronate revealed many endothermic peaks at 123.99 °C, 148.22 °C, 188.56 °C, and 198.53 °C. These peaks represent the gradual dehydration, as risedronate is a water-soluble drug containing a large amount of water known as hemipentahydrate (Redman-Furey et al., 2005). The thermal analysis of NFC showed a characteristic peak at 78.50 °C, which indicates the evaporation of water. Chitosan showed a broad peak at 105.27 °C, as a result of dehydration due to the weak hydrogen bonds between water and chitosan molecules (Zawadzki & Kaczmarek, 2010; Maged et al., 2019). The thermogram of citric acid demonstrated three endothermic peaks at 62.47 °C, 141.51 °C, and 170.16 °C. The peak below 100 °C indicates the water loss during the dehydration process; the second one represents the melting of citric acid, and is followed by decomposition occuring at the highest temperature into aconitic acid and citraconic acid anhydride (Sadik et al., 2018). The DSC analysis of the SC-T3 aerogel scaffold showed the formation of a new endothermic peak at 201.97 °C and the absence of the characteristic peaks of the individual components. This result confirms the generation of a new biopolymeric structure due to the cross-linking between chitosan and NFC.

#### 3.2.6. Scanning electron microscopy (SEM)

**Figure 5.**
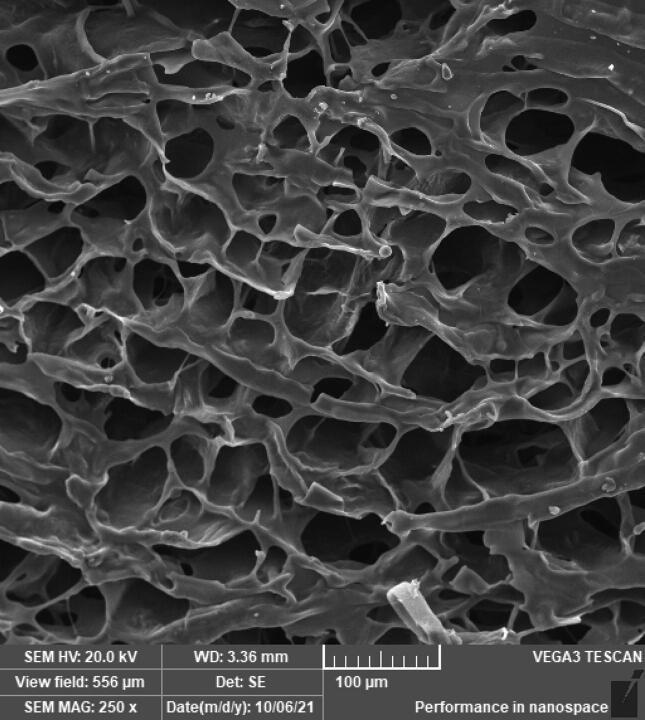
SEM photograph of the selected aerogel scaffold (SC-T3).

Figure 5 shows the SEM image of the selected aerogel scaffold (SC-T3). The highly porous microstructure with interconnecting channels is clear and confirms the porosity results measured using the helium pycnometer.

#### 3.2.7. Cell biology study

**Figure 6.**
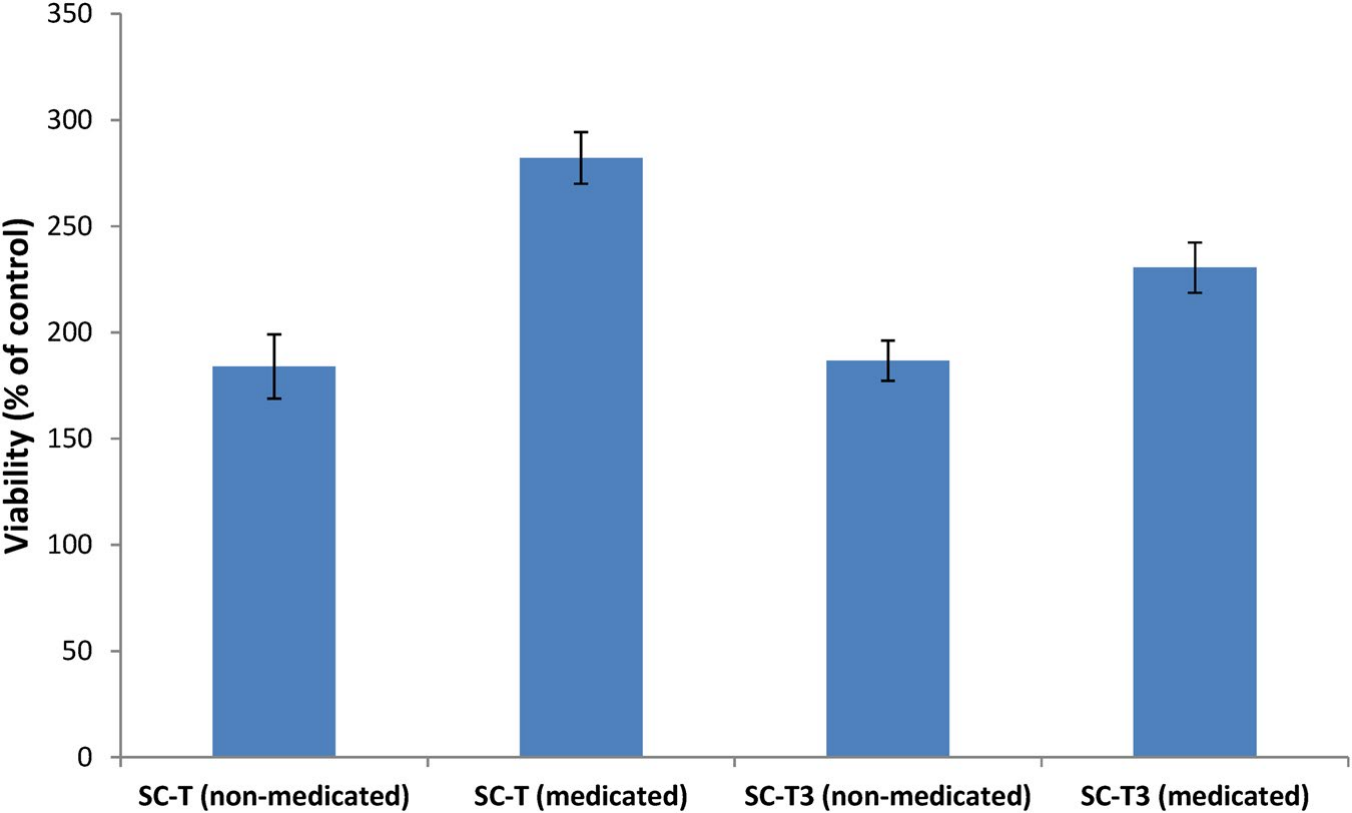
Cell viability results showing a significant increase in the case of all the tested samples compared to the control (p˂0.05).

The ability of the selected aerogel scaffold to regenerate bone was evaluated using the MG-63 cell line (human osteosarcoma fibroblasts). Medicated and non-medicated SC-T and SC-T3 aerogel scaffolds were tested for their ability to increase the proliferation of MG-63 cells. All the prepared aerogel scaffolds revealed a significant increase in cell viability compared to the control (*p* < 0.05) (Figure 6). Medicated aerogel scaffolds (SC-T and SC-T3) succeeded to increase cell growth compared to their counterpart non-medicated aerogel scaffolds (*p* < 0.05). Although the SC-T3 aerogel scaffold contains chitosan, which enhances cell regeneration and growth, it showed a lower proliferative activity on MG-63 cells than the SC-T aerogel scaffold (*p* < 0.05).

**Figure 7.**
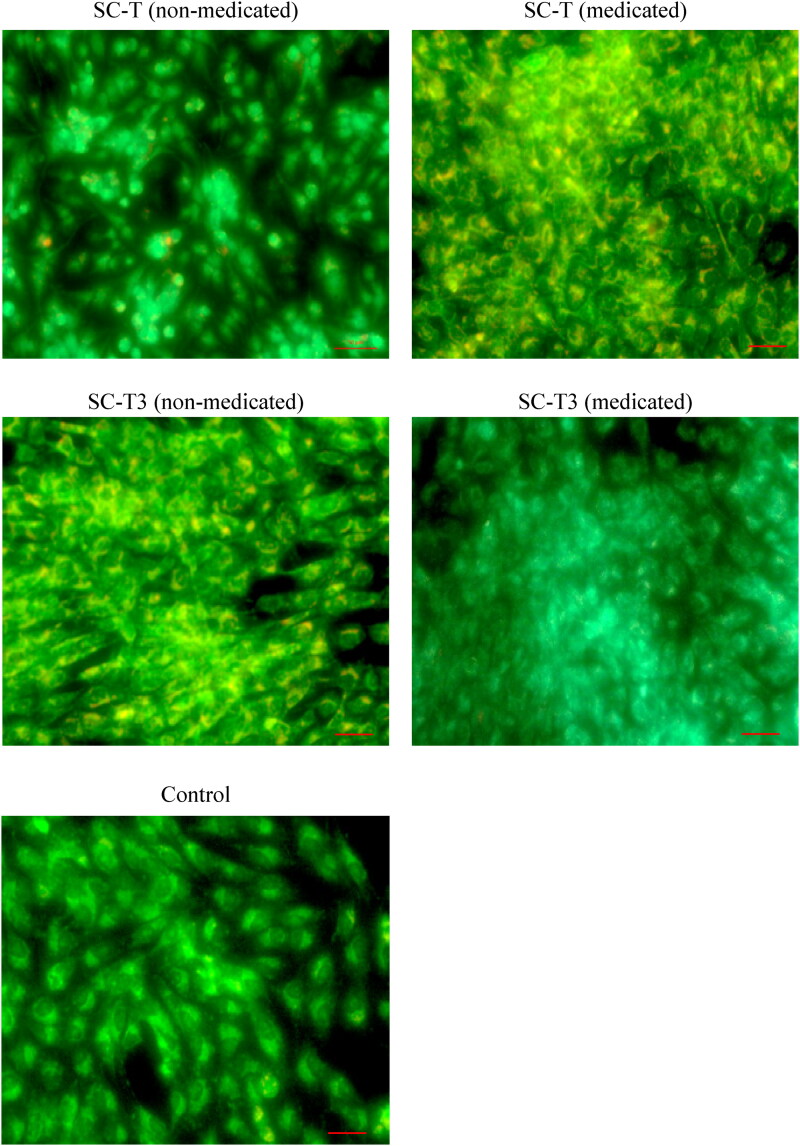
The mode of cell death using acridine orange/ethidium bromide stain on MG-63 cells. The photos show increase in cellular density for all tested samples in comparison to the control cells. (Magnification 20X, Scale bar 50 µm).

Figure 7 shows the mode of cell death using acridine orange/ethidium bromide stain on MG-63 cells. It is clear that all tested samples showed increased cellular proliferation in comparison to the control, with the medicated aerogel scaffolds having a higher regenerative ability than the corresponding non-medicated ones.

**Figure 8.**
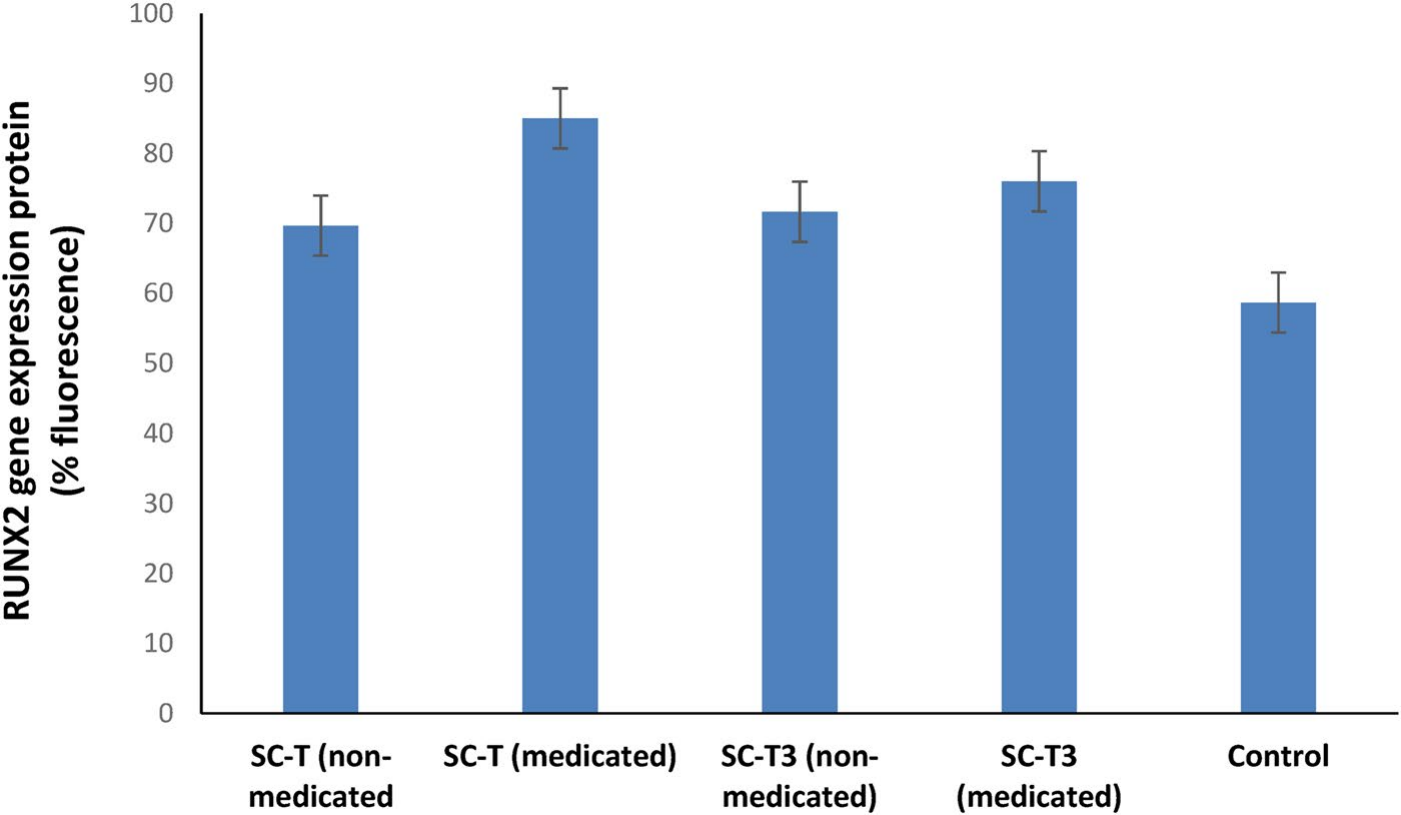
The RUNX2 protein expression results show a significant increase in RUNX2 protein expression in all tested samples compared to the control (p˂0.05).

**Figure 9.**
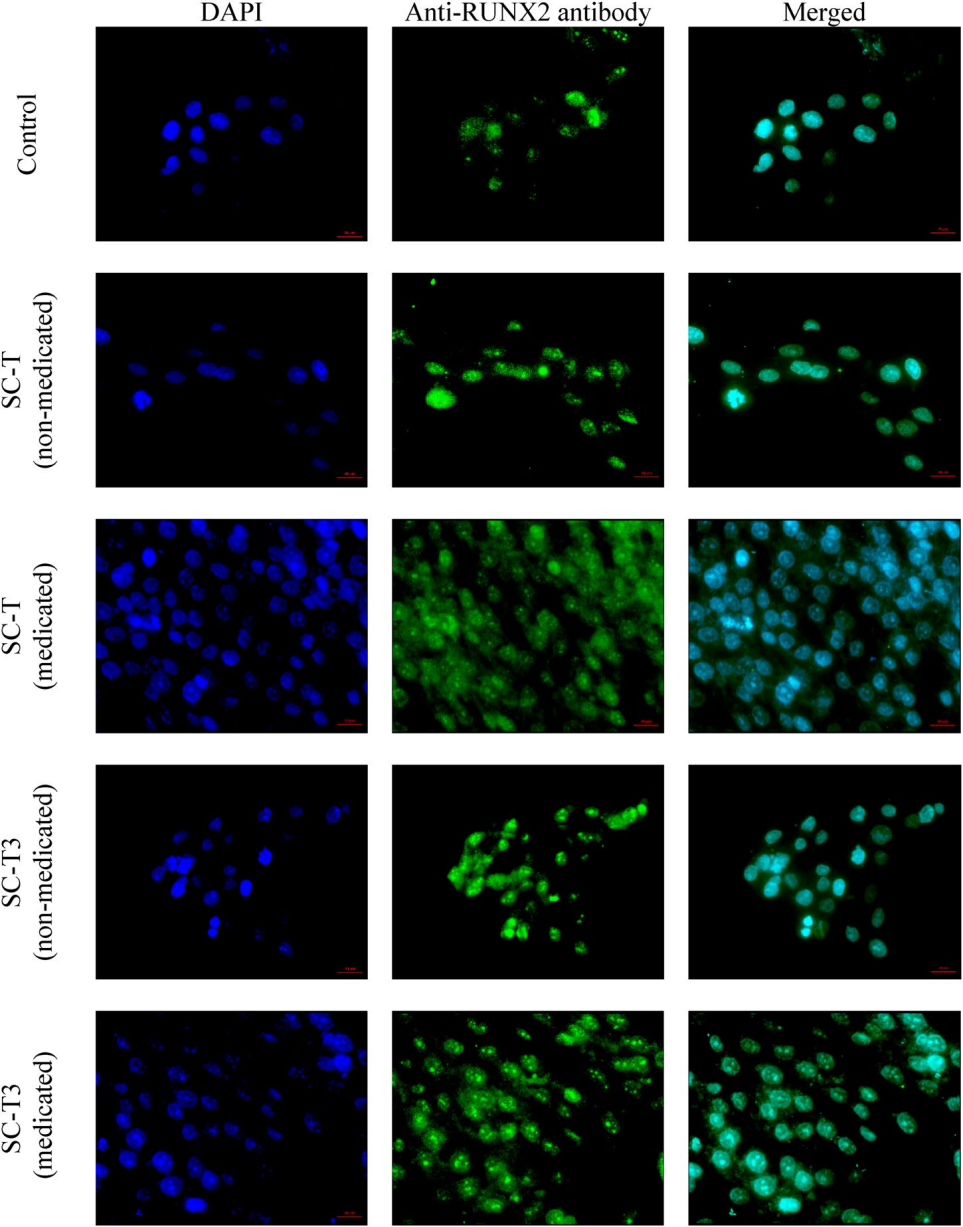
The photos show the pattern of RUNX2 protein expression for the tested samples in comparison to the control group. The RUNX2 protein is localized in the nucleoplasm. All the tested aerogel scaffolds showed a significant increase in the protein expression in comparison to the control group. (Magnification 40X, Scale bar 20 µm).

In addition, the RUNX2 protein expression data and photos (Figures 8 and 9) run in coincidence with the above displayed cell viability results. Bone maturation is dependent on osteoblast differentiation, and therefore, it is related to protein gene expression in the osteoblasts found in bone matrix. RUNX2 is known to be a key player in the regulation of bone development, maturation and maintenance by different mechanisms of action involving the function and differentiation of osteoblasts (Komori, 2008).

As a summary, the cell line study pointed the following results: (a) The increase in cell viability in the tested scaffolds compared to the control is attributed to the porosity created by the lyophilization process which supports water, nutrients, and cell adhesion inside the scaffold, enhancing cell differentiation and proliferation (Maged et al., 2019; Kamel et al., 2022a). Furthermore, cellulose is a biopolymeric material that reveals no toxicity and biocompatibility with living cells (Yahya et al., 2020). The cellular proliferative and reproductive effect of nanocellulose derived from agro-wastes has been highlighted in many studies proving its beneficial use as a pharmaceutical ingredient in the field of regenerative medicine (Kamel et al., 2020, 2022a,b). (b) Risedronate loaded in the aerogel scaffolds was able to increase cell viability as it is a bisphosphonate drug that inhibits bone resorption, supports osteogenic properties, and enhances the bone regeneration (Cheng et al., 2020). (c) The presence of citric acid in the chitosan-based formulation (SC-T3), might impair the mitochondrial pathways of living cells and affect the cell growth rate (Ying et al., 2013) and therefore, decrease cell growth. Also, the increased total solid content in SC-T3 compared to SC-T aerogel scaffolds, due to the addition of chitosan decrease the void spaces necessary for nutrients exchange, cellular adhesion and proliferation (Kamel et al., 2022a) which can be also the reason for decreasing the cell viability for SC-T3. This confirms the previous reported results regarding the cell proliferative effect of the aerogel scaffolds’ components in addition to its physicochemical properties enhancing cellular growth and adhesion.

## 4. Conclusion

Amorphous cellulose (AmC), TEMPO-oxidized cellulose (NFC) and citric acid-cross-linked cellulose (NFC/AmC) nanofibers were prepared in this study. The properties of these nanofibers were examined using FTIR, X-ray diffractometry, and DSC. NFC and NFC/AmC showed dense and porous structures, while AmC showed an agglomerated, loose structure. The rigidity of NFC/AmC, due to its cross-linked structure, resulted in the formation of aerogel scaffolds with high brittleness and low compressibility compared to those prepared using NFC. All the fabricated aerogel scaffolds showed high porosity (above 90%). The addition of chitosan to NFC scaffolds slowed down the release of risedronate from them. The selected aerogel scaffolds prepared using NFC and chitosan were able to increase MG-63 cell proliferation and RUNX2 gene expression protein and, this effect increased when the scaffolds were loaded with risedronate. On the other hand, the presence of citric acid in chitosan-containing scaffolds decreased cell growth. These findings highlight the promising approach of using biopolymers produced from agro-wastes in pharmaceutical applications.

## Author statement

All authors: Conceptualization, data analysis and curation, manuscript writing and revision; Nahla El-Wakil, Ragab E. Abouzeid and Alain Dufresne: Studies related to cellulose preparation and characterization. Rabab Kamel, Azza A. Mahmoud and Amr Maged: Studies related to scaffolds preparation, characterization and in-vitro evaluation; Mahmoud T. Abo el-fadl: Cell biology studies. All authors read and approved the final manuscript.
